# The 30-s chair stand test can be a useful tool for screening sarcopenia in elderly Japanese participants

**DOI:** 10.1186/s12891-021-04524-x

**Published:** 2021-07-24

**Authors:** Shuji Sawada, Hayao Ozaki, Toshiharu Natsume, Pengyu Deng, Toshinori Yoshihara, Takashi Nakagata, Takuya Osawa, Yoshihiko Ishihara, Tomoharu Kitada, Ken Kimura, Nobuhiro Sato, Shuichi Machida, Hisashi Naito

**Affiliations:** 1 COI project center, Juntendo University, 2-1-1 Hongo, Bunkyo-ku, Tokyo, 113-8421 Japan; 2 School of Health and Sports Science, Juntendo University, 1-1 Hirakagakuendai, Inzai, Chiba, 270-1695 Japan; 3 School of Sport and Health Science, Tokai Gakuen University, 21-233 Nishinohora, Ukigai, Miyoshi, Aichi, 470-0207 Japan; 4 Department of Human Structure & Function, Tokai University School of Medicine, 143 Shimokasuya, Iesehara, Kanagawa, 259-1193 Japan; 5 Graduate School of Health and Sports Science, Juntendo University, 1-1 Hirakagakuendai, Inzai, Chiba 270-1695 Japan; 6 Department of Physical Activity Research, National Institutes of Biomedical Innovation, Health and Nutrition, 1-23-1 Toyama, Shinjuku-ku, Tokyo, 162-8636 Japan; 7 Faculty of Sports and Health Sciences, Japan Women’s College of Physical Education, 8-19-1, Kitakarasuyama, Setagaya-ku, Chiba Tokyo, 157-8565 Japan; 8 School of Science and Technology for Future Life, Tokyo Denki University, 5 Senju Asahi-cho, Adachi-ku, Tokyo, 120-8551 Japan; 9 Faculty of Business Administration, Seijoh University, 2-172 Fukinodai, Tokai City, Aichi, 476-8588 Japan; 10 School of Engineering, Tokyo Denki University, 5 Senju Asahi-cho, Adachi-ku, Tokyo, 120-8551 Japan; 11 Graduate School of Medicine, Juntendo University, 2-1-1 Hongo, Bunkyo-ku, Tokyo, 113-8421 Japan; 12 Institute of Health and Sports Science & Medicine, Juntendo University, 1-1 Hirakagakuendai, Inzai, Chiba, 113-8421 Japan

**Keywords:** Elderly, Sarcopenia, Lower limb muscle strength, Chair stand test

## Abstract

**Background:**

Low muscle strength has been focused on as an essential characteristic of sarcopenia, and the 30-s chair stand test (CS-30) could be a particularly useful test for assessing muscle strength. While it is speculated to be a beneficial tool for the assessment of sarcopenia, this remains to be verified. In this study, we examined the reliability and optimal diagnostic score of the CS-30 for assessing sarcopenia in elderly Japanese participants.

**Methods:**

This cross-sectional study included 678 participants (443 females and 235 males) who underwent the test for sarcopenia as per the Asian Working Group for Sarcopenia (AWGS) 2019, the CS-30 test, and the isometric knee-extension muscle strength test. ROC analysis was used to estimate the optimal CS-30 scores at which sarcopenia was detected.

**Results:**

CS-30 scores were positively associated with sarcopenia (OR: 0.88; 95% CI:0.82–0.93). The AUC of the CS-30 for sarcopenia definition were 0.84 (*p* < 0.001) for females and 0.80 (*p* < 0.001) for males. The optimal number of stands in the CS-30 that predicted sarcopenia was 15 for females (sensitivity, 76.4%; specificity, 76.8%) and 17 for males (sensitivity, 75.0%; specificity, 71.7%).

**Conclusions:**

The CS-30 was found to be a reliable test for sarcopenia screening in the elderly Japanese population.

## Background

It is estimated that the number of individuals over the age of 60 years will rapidly increase in the coming decades [1]. As the elderly population continues to expand, the importance of maintaining the health and life expectancy of elderly adults has become an increasing concern, and sarcopenia is one of the most important concerns. The term “sarcopenia” was first introduced in 1989 and was defined as age-related loss of muscle mass [2–4]. Subsequently, the European Working Group on Sarcopenia in Older People (EWGSOP) defined sarcopenia as a syndrome characterized by progressive and generalized loss of skeletal muscle mass and strength, and recommended assessment of muscle mass and muscle function, including strength and performance, for a conclusive diagnosis to be made [5]. Based on recommendations from the EWGSOP, the Asian Working Group for Sarcopenia (AWGS) proposed a diagnostic algorithm for diagnosis in Asian countries (AWGS 2014) [6].

Recently, both groups revised their definitions. In these new definitions (EWGSOP2 and AWGS 2019), low muscle strength was focused on as an essential characteristic of sarcopenia, and the chair stand test was indicated as an effective test for assessing muscle strength or physical performance [7, 8]. It was specifically reported that the five-times sit-to-stand test (5STS) could be utilized as a simple and valuable tool in sarcopenia screening [9]. In this test, a subject is asked to sit and then stand repeatedly for five times as quickly as possible. The recommended 5STS cut-off time used to distinguish sarcopenia by the EWGSOP2 is > 15 s. The recommended AWGS 2019 diagnostic time is ≥ 12 s. These cut-off times were derived from previous studies that reported on the association of the 5STS and physical performance [10, 11].

While a previous study revealed the effectiveness of the 5STS in sarcopenia screening [9], further studies found that 21.6–26% of community-dwelling elderly adults could not complete the test [12, 13]. To solve this problem, we focused on a timed variation of the chair stand test as a more inclusive diagnostic tool in the elderly population. This variation scores the number of stands a participant can perform in a certain period. The 30-s chair stand test (CS-30) was previously reported to be useful for evaluating lower muscle strength in community-dwelling elderly participants [14] and was validated in elderly Japanese participants [15]. As described above, low muscle strength has been focused on as an essential characteristic of sarcopenia. While muscle strength can be assessed using the weight bearing index (WBI: quadriceps muscle strength/body weight), which is measured by maximum isometric strength of knee extension as described previously [16], muscle strength measurement devices are expensive and large [17]. Thus, the CS-30 could be a particularly useful test in assessing muscle strength. While it is speculated that it could also be a beneficial tool for the assessment of sarcopenia, this remains to be verified. In this study, we aimed to verify the relationship between the CS-30 test and sarcopenia diagnosis in elderly Japanese participants.

## Methods

### Participants

The study participants included healthy, elderly, community-dwelling Japanese participants. All participants were recruited through printed media, such as recruitment flyers or posters that were distributed or displayed in community or public facilities, and were informed of the methods, procedures, and risks of the study. They provided written informed consent prior to participating in study. We excluded individuals who did not follow our instructions or those with medical conditions that the physician in charge considered capable of limiting their ability to participate in the test. A total of 678 participants (443 females and 235 males) aged 74.7 ± 7.2 years (range, 65–97 years) to participate in this study.

Height, weight, appendicular skeletal muscle mass (ASM), grip strength, gait speed, and lower-limb muscle strength were evaluated. This study was conducted in accordance with the principles of the Declaration of Helsinki and was approved by the Ethics Committee for Human Experiments of Juntendo University Graduate School of Health and Sports Science, Chiba, Japan (Approval Number: 27–52).

### AWGS 2019-defined sarcopenia measurements

The algorithm and criteria of the AWGS 2019 were used to define sarcopenia in this study. Specifically, low muscle strength, low physical performance, and low muscle mass were used to diagnose sarcopenia, and the detailed criteria are described below.

Muscle strength: Handgrip strength was measured in the standing position with full elbow extension using a Smedley-type hand dynamometer (T.K.K.5401; Takei Kiki Kogyo, Niigata, Japan). The participants were cheered through verbal instruction. Two trials were completed with each hand, the tests were conducted by alternating between both hands, and the trial of the opposite hand was conducted during the rest period between each trial. The highest of the four values was used in the analysis. The handgrip strength measurement is the recommended method for detecting low muscle strength, and the cut-off criterion for low muscle strength was defined as < 28.0 kg for males and < 18.0 kg for females.

Physical performance: Gait speed was used to evaluate physical performance. It was calculated by measuring the time it took the study participants to walk across a 10 m corridor on a hard-surface floor. The participants were instructed to walk down the corridor at their usual speed, and they were given 2 m at the beginning and at the end of the 10-m corridor respectively, for acceleration and deceleration. Each participant completed two timed trials, and the average time was used in the analysis as per the AWGS 2019 recommendation. The cut-off criterion for low physical performance was defined as < 1.0 m/s for males and for females.

Muscle mass: Height squared-adjusted ASM (kg/m^2^) was assessed with multi-frequency bioimpedance analysis (MF-BIA) using a direct segmental multi-frequency bioelectrical impedance analyzer (InBody 730, InBody, Korea). Participants were instructed to sit calmly on a chair for 30 min before the measurement. The cut-off criterion for low muscle mass was < 7.0 kg/m^2^ for males and < 5.7 kg/m^2^ for females.

### Test measurements

Lower-limb muscle strength: Maximum isometric knee-extension muscle strength was measured using a tension meter (Takei, Tokyo, Japan). Participants were seated on a chair with their hip and knee joints flexed at 90° (0°, full hip or knee extension) and were instructed to exert an isometric force against the dynamometer by extending their knees 5 s [18]. Each participant underwent two or three trials and their highest result, normalized by body weight, was used to calculate their weight-bearing index (WBI).

CS-30 scores: Participants were instructed to complete sit-to-stand trials using a 40-cm high seat without using their arms as many times as possible in 30 s as outlined by Jones, 1999 [14]. The number of stands was recorded as their score.

### Statistical analysis

Shapiro–Wilk test was used to test for normality, and Spearman’s correlation analysis was used to determine the relationship between CS-30 scores and WBI, CS-30 scores and gait speed, and CS-30 scores and handgrip strength. The association between AWGS 2019-diagnosed sarcopenia and CS-30 score was analyzed by binary logistic regression analysis using the stepwise backward selection technique (*p*-value for inclusion and removal was 0.1). The CS-30 score, age, and gender were included as variables in the regression model, and the age- and sex-adjusted odds ratio (OR) was calculated [19]. To determine the optimal CS-30 cut-off score at which sarcopenia was said to occur, the parameters provided by the receiver operating characteristic (ROC) curve were analyzed by sex. The area under the curve (AUC), sensitivity, and specificity were calculated. The optimal CS-30 cut-off score was identified as the point on the curve closest to (0,1). A *p*-value of ˂0.05 was considered statistically significant.

Results are expressed as mean ± standard deviation (SD), and the OR is expressed as mean and 95% confidence interval (95% CI). Statistical analyses were performed using BellCurve for Excel (Social Survey Research Information Co., Ltd., Tokyo, Japan).

## Results

### Characteristics of participants

Table 1 shows the characteristics of the participants, and Table 2 shows the results of measurement items for diagnosing sarcopenia and CS-30. The average CS-30 score was 19.1 ± 6.2 stands (18.5 ± 6.4 stands for females, 20.1 ± 5.6 stands for males; Table 2), and it ranged from 0 to 37 repetitions. There were 5 persons whose CS-30 scores ranged from 0 to 4 repetitions.

**Table 1:** Characteristics of participants

	**All**	**Female**	**Male**
Age (years)	**74.7 ± 7.2**	**74.8 ± 7.5**	**74.5 ± 6.7**
Height (cm)	**156.4 ± 8.5**	**151.7 ± 5.7**	**165.2 ± 5.5**
Weight (kg)	**55.1 ± 9.9**	**50.7 ± 7.6**	**63.4 ± 8.3**
BMI (kg/m^2^)	**22.4 ± 2.9**	**22.0 ± 3.0**	**23.2 ± 2.7**

Values are means ± standard division (SD). *BMI* body mass index

**Table 2:** Results of measurement items for diagnosing sarcopenia and CS-30

	**All**	**Female**	**Male**
Handgrip strength (kg)	**27.2 ± 8.1**	**22.7 ± 4.5**	**35.8 ± 6.2**
Gait speed (m/s)	**1.32 ± 0.24**	**1.30 ± 0.25**	**1.35 ± 0.21**
Height squared-adjusted ASM (kg/m^2^)	**6.31 ± 1.01**	**5.76 ± 0.67**	**7.35 ± 0.64**
CS-30 (stands)	**19.1 ± 6.2**	**18.5 ± 6.4**	**20.1 ± 5.6**

Values are means ± standard division (SD). *ASM* appendicular skeletal muscle mass; CS-30, the 30-s chair stand test

### The relationship between sarcopenia and CS-30

**Fig. 1:**
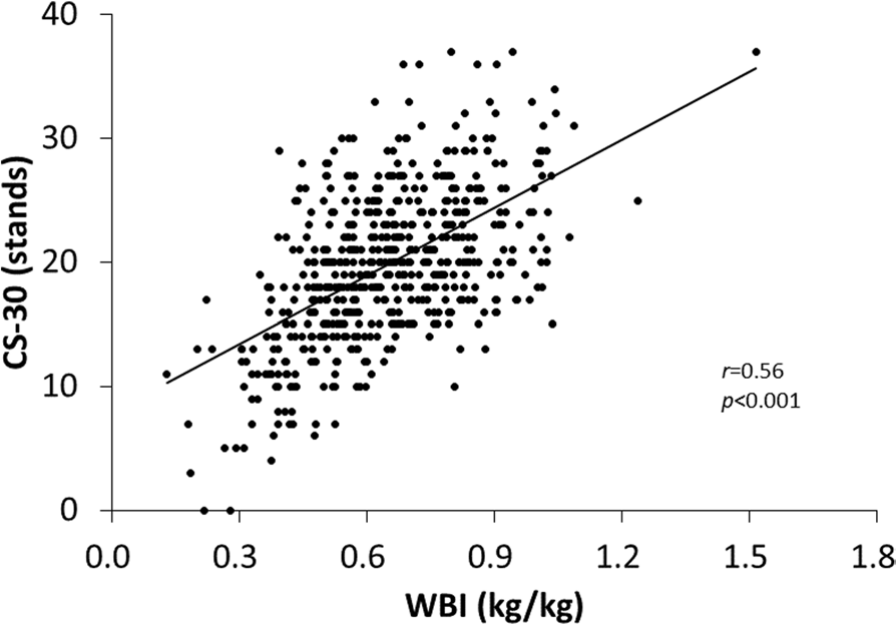
Correlation between WBI and CS-30. Data were analyzed using correlation analysis. N = 678

**Fig. 2:**
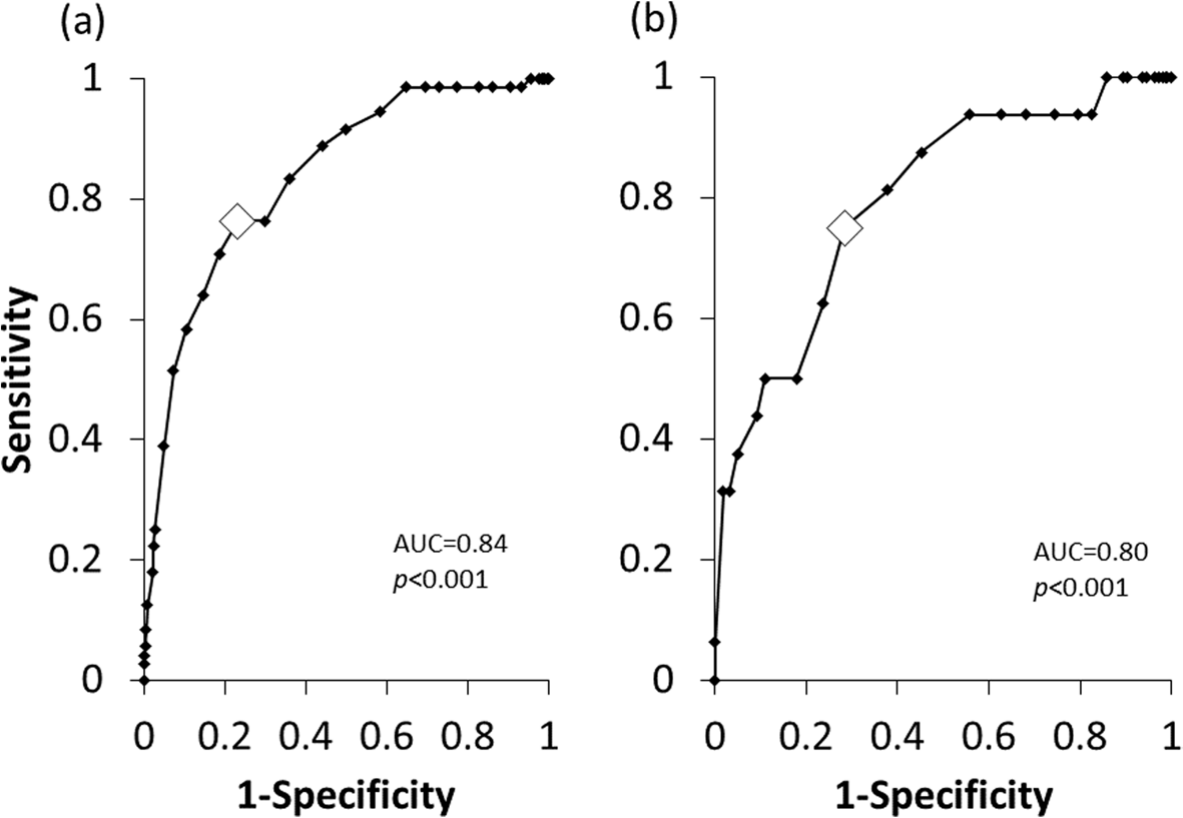
ROC of CS-30 for discriminating sarcopenia in females (**a**) and males (**b**). Data were analyzed using ROC analysis. N = 678

The CS-30 score was significantly correlated with WBI (*r* = 0.53, *p* < 0.001; Fig. 1), gait speed (*r* = 0.57, *p* < 0.001), and handgrip strength (*r* = 0.40, *p* < 0.001). Binary logistic regression showed that a higher CS-30 score significantly decreased the odds of having sarcopenia (OR: 0.88; 95% CI:0.82–0.93; *p* < 0.001; Table 3). ROC curve analysis indicated that the AUC for female participants was 0.84 (*p* < 0.001; Table 4). Based on the analysis, the optimal CS-30 cut-off score for differentiating sarcopenia diagnosis in females was found to be 15 stands (sensitivity, 76.4%; specificity, 76.8%) (Fig. 2a). In male participants, the AUC was 0.80 (*p* < 0.001; Table 4) and the optimal CS-30 cut-off score was 17 stands (sensitivity, 75.0%; specificity, 71.7%) (Fig. 2b).

**Table 3:** Result of the binary logistic regression analysis

	**Unadjusted Model 0**				**Multivariable Model 1**^**a**^			
	**OR** **(95% CI)**	**Cox-Snell R**^**2**^	**Nagelkerke R**^**2**^	***p***	**OR** **(95% CI)**	**Cox-Snell R**^**2**^	**Nagelkerke R**^**2**^	***p***
**CS-30**	**0.77** **(0.73–0.82)**	**0.162**	**0.306**	** < 0.001**	**0.88** **(0.82–0.93)**	**0.213**	**0.403**	** < 0.001**

^a^: Age and gender adjusted

CS-30, the 30-s chair stand test; OR, odds ratio; CI, confidence interval

**Table 4:** Comparison between CS-30 and 5STS

		**AUC for sarcopenia**	**Correlation with gait speed**	**Correlation with WBI**
**CS-30**	**Female**	**0.84**	**0.60**	**0.56**
	**Male**	**0.80**		
**5STS**	**Female**	**0.72**^**(9)**^	**-0.58**^**(11)**^	**-0.43**^**(19)**^
	**Male**	**―**		

Correlation coefficients are described

*AUC* the area under the curve, *WBI* weight-bearing index; *5TST* the five-times sit-to-stand test, *CS-30* the 30-s chair stand test

## Discussion

The examination of the linear relationship between the CS-30 and WBI revealed a mild correlation between the two tests (Fig. 1). A similar correlation was found in a previous study of 306 community-dwelling elderly Japanese participants (mean age 73.6 ± 6.6 years) [20]. Additionally, the correlation between the 5STS and WBI was also found in a previous study of 669 community-dwelling men and women aged 75–93 years (mean age 78.9 ± 4.1 years) [21]. Although the assessment of WBI can be a relevant method for assessing lower-limb muscle strength, dedicated machinery was required to conduct the test, and the risk of injury is much greater than that of the sit-to-stand test for elderly individuals. The positive correlation between CS-30 and WBI was indicative of the CS-30’s assessment capabilities for lower-limb muscle strength.

A previous study that assessed the ability of the 5STS to diagnose sarcopenia in elderly female participants revealed an AUC of 0.72 [9]. The AUC in the present study was found to be 0.84 in females and 0.80 in males (Table 4). In addition to identifying the CS-30 as a discriminatory test for sarcopenia, the optimal CS-30 cut-off scores determined in this study can be assigned as a simple and meaningful goal for elderly people to increase their lower-limb muscle strength. The cut-off score for diagnosis indicates that people should be able to complete one sit-to-stand cycle at least every 2 s for 30 s to maintain lower muscle strength. From this point of view, we confirm that the CS-30 is a useful tool for screening the risk of AWGS 2019-defined sarcopenia, as predicted.

Our findings show that chair stand test can be a reliable method for screening the risk of sarcopenia, and this is consistent with the findings of previous studies. On the other hand, the CS-30 cut-off score of 15 stands in females and 17 stands in males for making a diagnosis of sarcopenia indicates that even an individual with a 5STS of < 12 s might still be at risk of sarcopenia. From this point of view, our findings are novel as this study assessed whether the CS-30 is useful in the early detection of sarcopenia.

This study has a few limitations. First, we did not evaluate the 5STS and CS-30 in the same group of participants; therefore, we cannot directly compare these methods. As the 5STS has been identified by AWGS 2019 as a recommended assessment tool for physical performance, it may be useful currently, to assess the CS-30 in addition. Second, there were only three variables in the binary logistic regression model, having a possibility of making the data of this study unsuitable for extrapolation to a wider population.

To overcome these limitations, future studies are required to compare the diagnostic reliability of the 5STS and the CS-30 for sarcopenia in the same group of participants, and to confirm the reliability of the CS-30 for screening the risk of AWGS 2019-defined sarcopenia in a wider population.

## Conclusions

In conclusion, the CS-30 was found to be a beneficial diagnostic tool for assessing the risk of sarcopenia in elderly Japanese participants. Optimal CS-30 cut-off scores were established and a positive correlation between the CS-30 and AWGS 2019-defined assessment methods was established.

## Data Availability

The datasets analyzed during the current study are available from the corresponding author on reasonable request.

## Abbreviations

CS-30: The 30-s chair stand test
5TST: The five-times sit-to-stand test
BMI: Body mass index
ASM: Appendicular skeletal muscle mass
WBI: Weight-bearing index
AUC: The area under the curve
ROC: The receiver operating characteristic
SD: Standard division
OR: Odds ratio
CI: Confidence interval
